# Development of a glycoproteomic strategy to detect more aggressive prostate cancer using lectin-immunoassays for serum fucosylated PSA

**DOI:** 10.1186/s12014-019-9234-4

**Published:** 2019-04-06

**Authors:** Ce Wang, Naseruddin Höti, Tung-Shing Mamie Lih, Lori J. Sokoll, Rui Zhang, Zhen Zhang, Hui Zhang, Daniel W. Chan

**Affiliations:** 1 Department of Pathology, Johns Hopkins Medicine, Smith Bldg 4013, 400 N. Broadway, Baltimore, MD 21287 USA; 2 Present Address: Department of Clinical Laboratory, Chinese People’s Liberation Army General Hospital, 28 Fuxing Road, Beijing, 100853 China

**Keywords:** Lectin-immunoassays, Glycoproteomic, Fucosylated PSA, Prostate cancer

## Abstract

**Background:**

Prostate-specific antigen (PSA) is commonly used as a serum biomarker for the detection of prostate cancer. However, levels of PSA in serum do not reliably distinguish aggressive prostate cancer from non-aggressive disease. Therefore, there is an urgent need for biomarkers that can differentiate aggressive prostate cancers from non-aggressive phenotypes. Fucosylation is one of the glycosylation-based protein modifications. Previously we demonstrated increased levels of serum fucosylated PSA in patients with aggressive prostate cancer using lectin selection followed by PSA immunoassay.

**Methods:**

We developed two lectin-immunoassays, Lens culinaris agglutinin (LCA) and Aleuria aurantia lectin (AAL) followed by clinical PSA immunoassay and investigated the levels of PSA and its fucosylated glycoforms in serum specimens from prostate cancer patients with different Gleason scores. First, we developed standard curves for lectins enrichment, which were applied to lectin-immunoassay for fucosylated PSA–LCA and PSA–AAL quantification in serum samples.

**Results:**

Our results showed that both LCA- and AAL-immunoassays detected elevated fucosylated PSA and were correlated with higher Gleason scores but only AAL-immunoassay detected an increased percentage of fucosylated PSA in patient serum with higher Gleason scores.

**Conclusion:**

We have developed quantitative lectin-immunoassays for serum fucosylated PSA. Our data demonstrated that fucosylated PSA–AAL, % fucosylated PSA–AAL and fucosylated PSA–LCA levels could be effective biomarkers to differentiate aggressive prostate cancer [especially Gleason 7 (4 + 3) or above] from non-aggressive disease. We believe that application of these lectin-immunoassays to a larger patient population is needed to evaluate the clinical utilities of fucosylated PSA using AAL–PSA and LCA–PSA for aggressive prostate cancer.

## Background

Prostate cancer (PCa) is the most common cancer in men in the United States [1]. In the past decade, the incidence of PCa continues to increase and has become the second leading cause of cancer death in men in the United States [2]. Clinically, more than 80% of men with aggressive (AG) PCa will develop skeletal complications which cause a dramatic reduction in quality of life [3]. Currently, Gleason score which is based on the architecture of prostate tissue is used to diagnose AG PCa [4, 5]. Cancers with lower Gleason scores (< 7) tend to be less aggressive, while cancers with higher Gleason scores (7 or above) tend to be more aggressive [6]. Gleason score 7 are reported as being either Gleason score 3 + 4 (with Gleason pattern 3 being more predominant with some Gleason pattern 4) or Gleason score 4 + 3 (with more predominant Gleason pattern 4 and some Gleason pattern 3). Most recent studies have shown that Gleason score 4 + 3 cancers tend to represent more severe disease, with higher risk of biochemical recurrence compared to Gleason scores 3 + 4 disease [7–9]. Prostate specific antigen (PSA) is a commonly used serum biomarker for early detection of PCa [10–12]. With the widespread introduction of PSA testing, more prostate cancers were discovered at early stages [13, 14]. However, PSA testing has limitations such as low specificity, false positive results and over-diagnosis [15, 16]. Some benign prostate diseases, especially benign prostatic hyperplasia (BPH) and inflammation of the prostate can also cause significant increases in serum PSA levels [17, 18]. In addition, PSA is less effective in distinguishing AG from non-aggressive (NAG) PCa and other benign prostatic diseases [19, 20]. Therefore, there is a need for biomarkers that can distinguish AG from the NAG PCa phenotypes and other benign conditions.

Glycosylation is one of the common protein modifications [21]. Several studies have shown aberrant glycosylation especially fucosylation (Fuc) in tumor biology [22–25]. For example, the fucosylated glycoform of α feto-protein (AFP-L3) gained the approval by the Food and drug administration (FDA) as a biomarker for the risk of developing hepatocellular carcinomas [26]. Aberrant Fuc is caused by alternations in glycosylation-related enzymes such as fucosyl-transferases (FUTs) and fucosidase [27, 28].

There have been several reported strategies for the analysis of fucosylated glycoproteins using lectins. Lectins are proteins that preferentially recognize oligosaccharide epitopes. Lectins have been widely used to selectively enrich glycosylated proteins and peptides to profile unique glycoforms [24, 29, 30]. Lectins that have affinity for binding to fucosylated glycoconjugates provide a selection method for glycoproteins containing fucosylated glycans. Both Aleuria aurantia lectin (AAL) and Lens Culinaris Agglutinin (LCA) have been used to detect fucosylated PSA [25, 31]. Previously, we found that LCA binds preferentially to the core Fuc (α 1-6 fucosylation), whereas AAL has affinity towards both core Fuc and branched Fuc (α 1-3/α 1-4 fucosylation) [25, 31].

In this study, we developed two lectin-based immunoassays for selection of fucosylated PSA followed by clinical PSA immunoassay. This glycoproteomic strategy is based on lectin-specific selection for fucosylated glycoproteins followed by PSA measurement using immunoassay. Using these assays, we evaluated the levels of fucosylated PSA bound by LCA and AAL and found that both LCA and AAL-immunoassays demonstrated a strong association between fucosylated PSA and Gleason score in PCa patients. However, the elevated percentage of fucosylated PSA was only detected by AAL–PSA assay. Our result demonstrated the utility of developing lectin-immunoassays in analyzing serum fucosylated PSA.

## Materials and methods

### Chemicals and reagents

Agarose bound AAL and LCA were purchased from Vector Labs (Burlingame, CA). BCA protein assay kits were purchased from Bio-Rad(Hercules, CA).

### Serum sample collection

24 serum samples from men with histologically determined PCa were studied. Serum samples were centrifuged at 5000*g* for 10 min after they were thawed to remove debris before processing for lectin selection. Each serum sample underwent no more than three freeze/thaw cycles prior to the test. The use of clinical samples was approved by the Johns Hopkins Institutional Review Board. All study cases were annotated with available clinical information in a manner that protected patient identities.

### Fucosylated glycoproteins selection by lectins

Agarose bound lectin beads (200 µg) were washed three times with 500 µL of TBS buffer (pH 7.4, Tris Buffered Saline) using centrifugation. 100 µL serum samples, beads and TBS buffer were then mixed overnight at 4 °C. After incubation, the beads were washed four times with 500 µL of TBS buffer to remove non-specific bindings. Then the Fuc glycoproteins were eluted with 200 µL of 200 mM Methyl ɑ-d-mannopyranoside and 200 mM Methyl α-d-glucopyranoside (for LCA elution) or 200 µL of 100 mM l-fucose (for AAL elution) in TBS buffer by shaking for 1 h. Lectin bound sample was collected by centrifugation.

### Standard curve of lectin enrichment

The PSA level of a serum pool was first identified using a clinical PSA immunoassay (Access 2 Hybritech PSA assay, Beckman Coulter, Brea, CA), then the serum pool was diluted to the following concentrations: 80, 40, 20, 10, 5, 2.5, and 0 ng/mL with the PSA assay diluent. For each PSA concentration, we used LCA and AAL lectins to enrich fucosylated PSA as described before [25, 31]. After elution, we detected the level of fucosylated PSA. Each data point was analyzed 5 times and the PSA diluent (Beckman Coulter, Brea, CA) was used as blank control. The PSA levels after lectin enrichment from different serum samples were used to form the standard curve.

### Lectin-immunoassay for Fuc PSA–LCA and PSA–AAL quantification in serum samples

100 µL of serum was diluted with PSA diluent at 1:2 ratios to a total volume of 200 µL. The protein concentration of serum was determined using Pierce™ BCA Protein Assay Kit (Thermo Fisher Scientific Inc, MA, USA). Agarose LCA and AAL lectins were incubated with protein in serum at 1:1 ratios in a microfuge tube, then fucosylated PSA in serum was enriched as previously described [31, 32].

### Data analysis

The percentage of fucosylated PSA was calculated using the value of individual fucosylated glycoproteins and the total PSA value before lectin selection. The statistical analyses and multiple logistic regression were done using SPSS Statistics version 17.0 (SPSS, Chicago, IL, USA) and GraphPad Prism 5 (GraphPad Software, La Jolla, CA, USA). Correlation analysis of the two lectins was done using Spearman analysis. The Kruskal–Wallis and Mann–Whitney tests were used to analyze the differences among different Gleason score categories [Gleason scores of 6, 7 (3 + 4 or 4 + 3), or 8–9]. The predictive power of fucosylated PSA and the percentage of fucosylated PSA were assessed using receiver operating characteristics (ROC) curve. The value of area under curve (AUC) was calculated as an indication of the accuracy of prediction. The ROC curves were generated and compared using MedCalc. All *p* values were two-sided, with statistical significance at *p* < 0.05.

## Results

### Development of fucosylated PSAassays using lectin enrichment

**Fig. 1:**
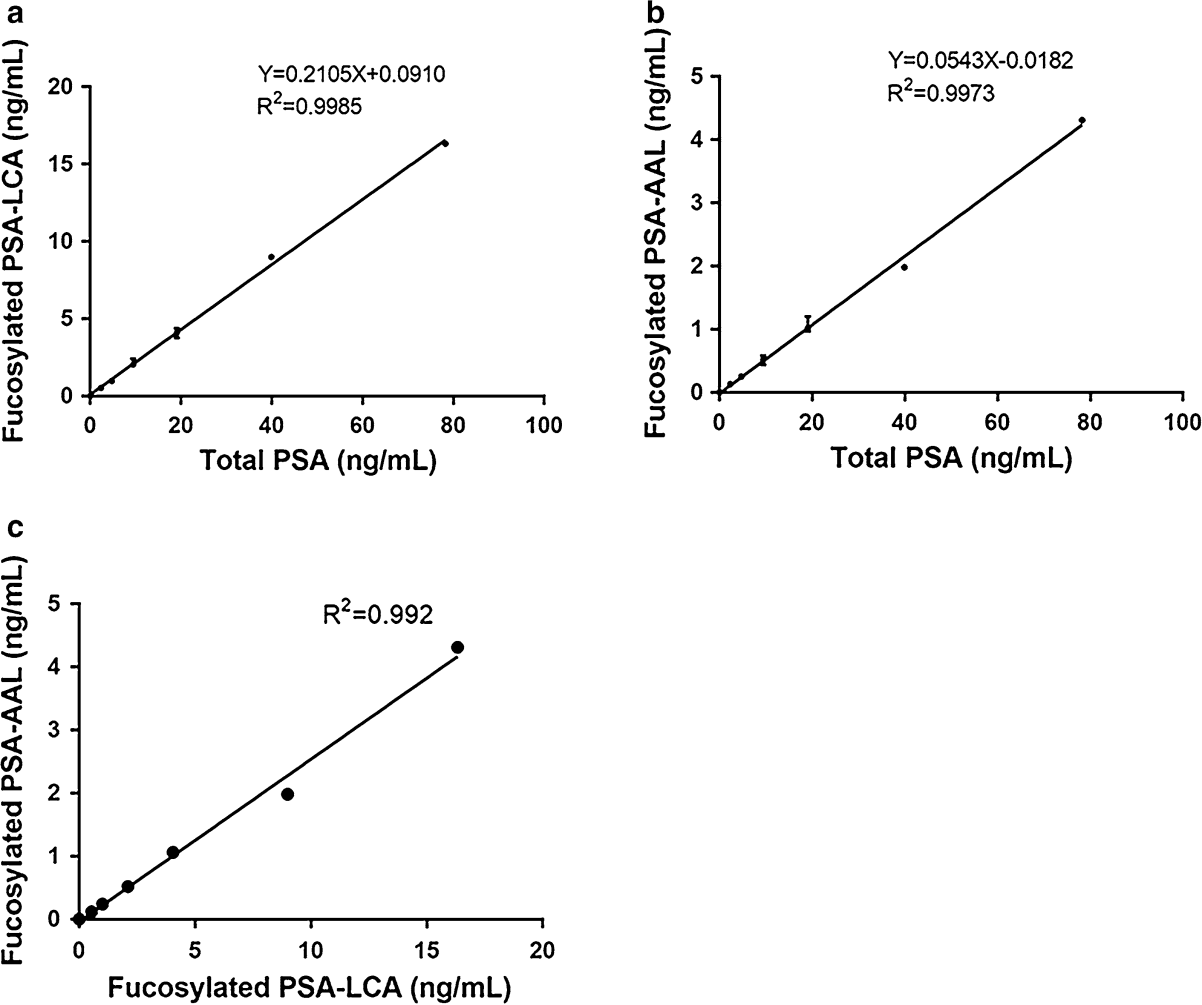
The standard curve of lectin-PSA immunoassays. **a** Linear range for LCA–PSA. **b** Linear range for AAL–PSA. **c** Comparison of LCA–PSA and AAL–PSA for the same level of PSA

To determine whether serum fucosylated PSA could be used to detect AG PCa, we developed fucosylated PSA assays using lectin selection and quantitation of fucosylated PSA with the clinical Access Hybritech PSA assay [32]. In our previous study, we found that LCA and AAL had differential binding affinities to core- and branch-Fuc forms of proteins respectively [31]. To determine the performance of fucosylated PSA assays, both LCA and AAL were used to select fucosylated PSA followed by a clinical PSA test. A series of dilution of serum pool with different PSA levels were enriched by LCA and AAL respectively. The serum total PSA and fucosylated PSA levels were quantified using the clinical Access Hybritech PSA assay to obtain a standard curve. As shown in the Fig. 1, the X-axis represents the total PSA level and the Y-axis represents the fucosylated PSA level. Each data point was done in 5 replicates. As shown in Fig. 1a, b, a linear trend of increasing fucosylated PSA level was observed against the total PSA at each data point.

Both fucosylated PSA assays using LCA and AAL showed the linear relationship between the fucosylated PSA level and total PSA level in the range of 0–80 ng/mL of total PSA (Y = 0.2105X + 0.0910, R^2^ = 0.9985; Y = 0.0543X − 0.0182, R^2^ = 0.9973; respectively). We observed the average % fucosylated PSA (the percentage of fucosylated PSA to total PSA) with LCA enrichment to be 21.05% and the average % fucosylated PSA with AAL enrichment to be 5.43%. We further compared the fucosylated PSA level using LCA or AAL enrichment at the same PSA levels. As shown in Fig. 1c, for the same PSA level, we can detect higher fucosylated PSA using LCA–PSA enrichment compared to AAL–PSA.

### Clinical characteristics of serum samples

Clinical characteristics of serum samples from patients with histologically-proven PCa were analyzed in our study. As shown in Table 1, among these serum samples, 33.3% cases (n = 8) were Gleason 6, 33.3% (n = 8) were Gleason 7, 12.5% (n = 3) were Gleason 8, and 20.8% (n = 5) were Gleason 9. Among Gleason 7, 20.8% (n = 5) were Gleason (3 + 4) and 12.5% (n = 3) were Gleason (4 + 3). The average age was 61.0 ± 8.0 years (range 43–79 years). There was no correlation between the Gleason score and age. The serum total PSA levels were determined using the Access Hybritech PSA assay were as follows, for Gleason 6 (median PSA: 4.2 ng/mL; range: 2.7-5.1 ng/mL), Gleason (3 + 4) (median PSA: 4.7 ng/mL; range: 2.8–6.8 ng/mL), Gleason (4 + 3) (median PSA: 8.2 ng/mL; range: 6.8–56.0 ng/mL), Gleason 8 (median PSA: 12.1 ng/mL; range: 8.1–14.0 ng/mL) and Gleason 9 (median PSA: 10.9 ng/mL; range: 3.7–25.9 ng/mL).

**Table 1:** Sample characteristics

	Gleason score at prostatectomy n (%)			Patients age (year)	Serum PSA level (ng/ml)
Pca (n = 24)	GS 6		8 (33.3%)	57.0 ± 7.0 (48.0–67.0)	4.2 ± 0.8 (2.7–5.1)
	GS 7	GS(3 + 4)	5 (20.8%)	60.0 ± 10.0 (43.0–67.0)	4.7 ± 1.8 (2.8–6.8)
		GS(4 + 3)	3 (12.5%)	62.0 ± 5.0 (62.0–66.0)	8.2 ± 28.1 (6.8–56.0)
	GS 8		3 (12.5%)	67.0 ± 4.0 (60.0–68.0)	12.1 ± 3.0 (8.1–14.0)
	GS 9		5 (20.8%)	63.0 ± 8.0 (59.0–79.0)	10.9 ± 9.2 (3.7–25.9)

### Serum fucosylated PSA levels analyzed by lectin-immunoassays

**Fig. 2:**
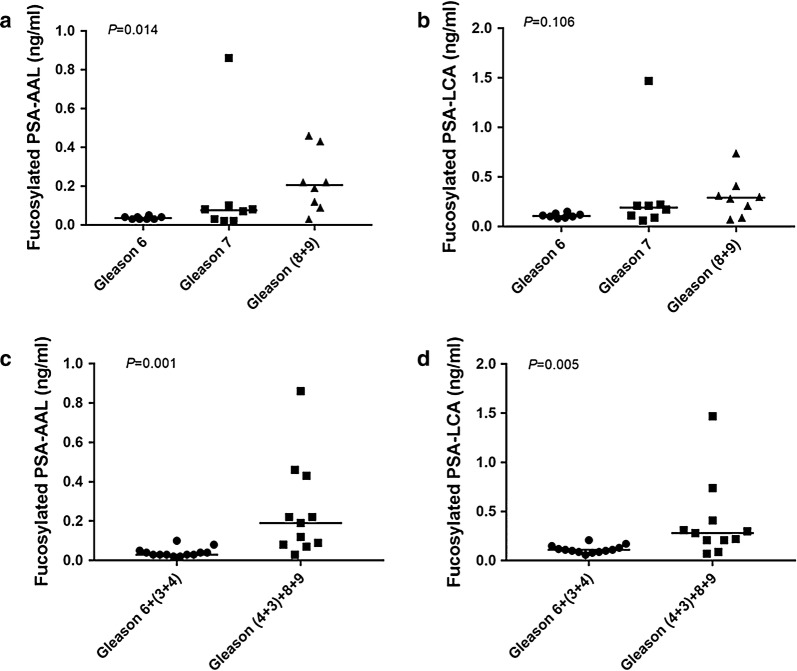
The correlation of serum levels of fucosylated PSA–AAL and fucosylated PSA–LCA with tumor Gleason scores. Serum fucosylated PSA after AAL selection with tumor Gleason scores are shown in (**a**, **c**). Serum fucosylated PSA after LCA selection with tumor Gleason scores are shown in (**b**, **d**). The lines indicate median values in each group

Applying the AAL–PSA and LCA–PSA assays to the prostate cancer serum samples, we observed significant differences in serum PSA levels among Gleason 6, Gleason 7 and Gleason 8, 9 for fucosylated PSA–AAL (Kruskal–Wallis; *p *= 0.014) (Fig. 2a). However, using the LCA lectin based PSA selection strategy, we did not find any differences among Gleason 6, Gleason 7 and Gleason 8, 9 (Kruskal–Wallis; *p *= 0.106) (Fig. 2b). The current PCa grading system is known to have several limitations, one of which is the heterogeneity of pathological Gleason 7 PCa which can be Gleason 3 + 4 or Gleason 4 + 3. It has been established from several studies that different outcomes are associated with Gleason 3 + 4 and Gleason 4 + 3. To evaluate the efficacy of the fucosylated form of PSA in distinguishing Gleason 6 (Gleason 3 + 3) and (Gleason 3 + 4) from Gleason 4 + 3 and Gleason 8 & 9, we stratified the Gleason 7 into two groups (Gleason 3 + 4, Gleason 4 + 3), then combined Gleason 7 (4 + 3) with Gleason 8, 9 into an AG PCa group, and Gleason 6 and 7 (3 + 4) into a NAG PCa group. Both serum fucosylated PSA–AAL and fucosylated PSA–LCA levels were found to be significantly higher in the group with a Gleason pattern of 7 (4 + 3) and above compared to the Gleason pattern 7 (3 + 4) and below (Mann–Whitney; *p *= 0.001 and *p *= 0.005, respectively) (Fig. 2c, d). Our results demonstrated that serum fucosylated PSA level were correlated with higher Gleason scores and aggressive phenotype of PCa.

### Percentage of serum fucosylated PSA using AAL–PSA and LCA–PSA immunoassays

**Fig. 3:**
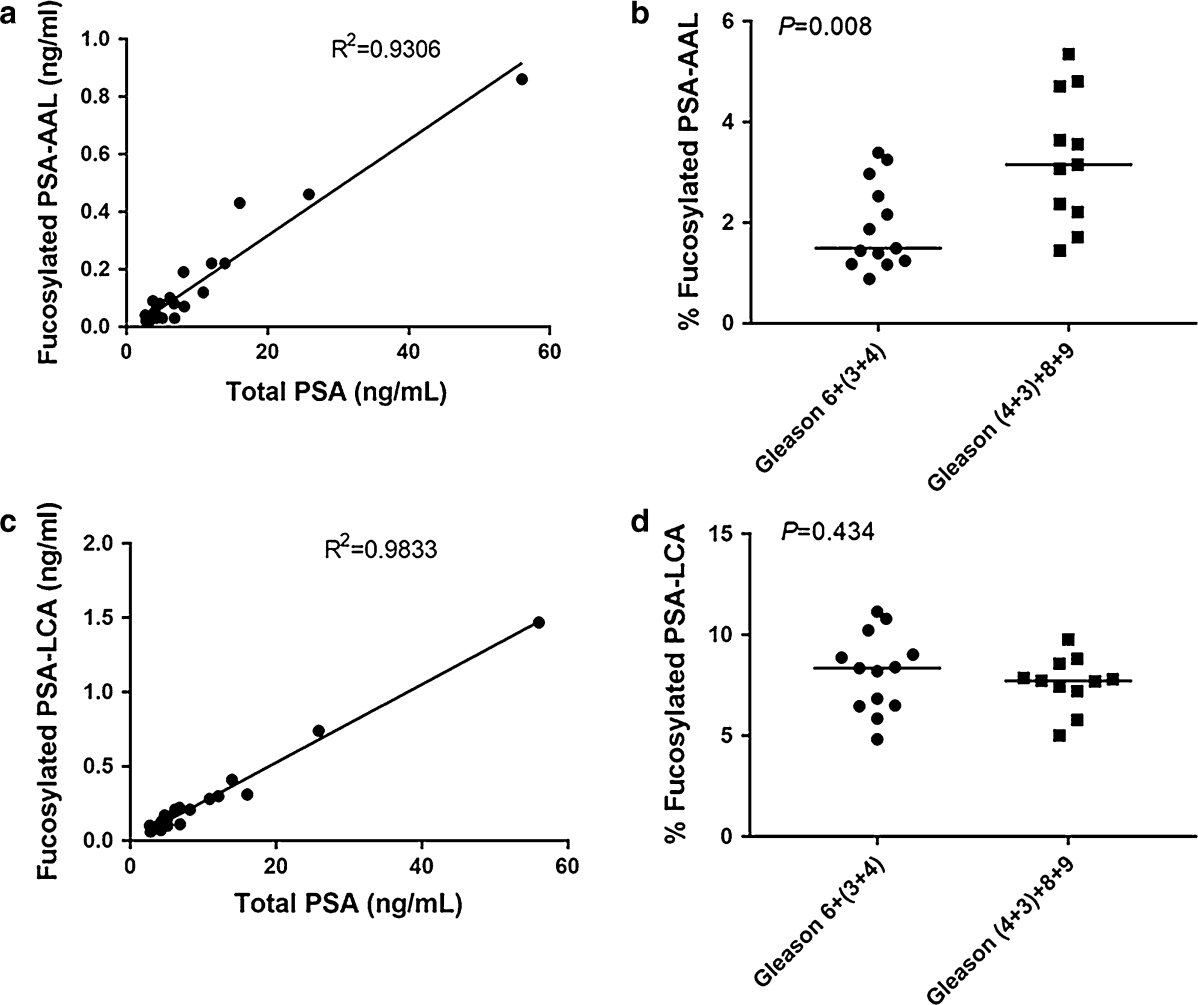
The correlation of serum levels of fucosylated PSA with total PSA and tumor Gleason scores. The correlation of serum fucosylated PSA level after lectin selection with total PSA are shown in (**a**, **c**). Serum % fucosylated PSA after lectin selection are shown in (**b**, **d**). The lines indicate median values in each group in (**b**, **d**)

To explore the relationship between serum total PSA and fucosylated PSA, we performed regression analysis. The correlation of total PSA to fucosylated PSA–AAL was 0.9306 (R^2^ in Fig. 3a) while the correlation of total PSA to fucosylated PSA–LCA was 0.9833 (R^2^ in Fig. 3c). Both fucosylated PSA–AAL and fucosylated PSA–LCA were highly correlated with total PSA levels, particularly fucosylated PSA–LCA. A significant difference in % fucosylated PSA–AAL was observed when we combined Gleason 6 and (3 + 4) into one group, and Gleason (4 + 3) and Gleason 8, 9 into another (Mann–Whitney; *p *= 0.008) (Fig. 3b), however,we did not find a significant difference in % fucosylated PSA–LCA (Mann–Whitney; *p *= 0.434) (Fig. 3d).

To further compare the utilities of total serum PSA and fucosylated PSA–AAL, we performed a multiple logistic regression analysis on the total PSA and fucosylated PSA between Gleason 6 and 7 (3 + 4) vs Gleason 7 (4 + 3) and Gleason 8, 9. Multiple logistic regression with a *p* value for total PSA (0.307) and fucosylated PSA–AAL (0.183) were obtained, which showed no obvious difference between total PSA and fucosylated PSA–AAL.

### Prediction of Gleason score by PSA and fucosylated PSA–AAL levels

**Fig. 4:**
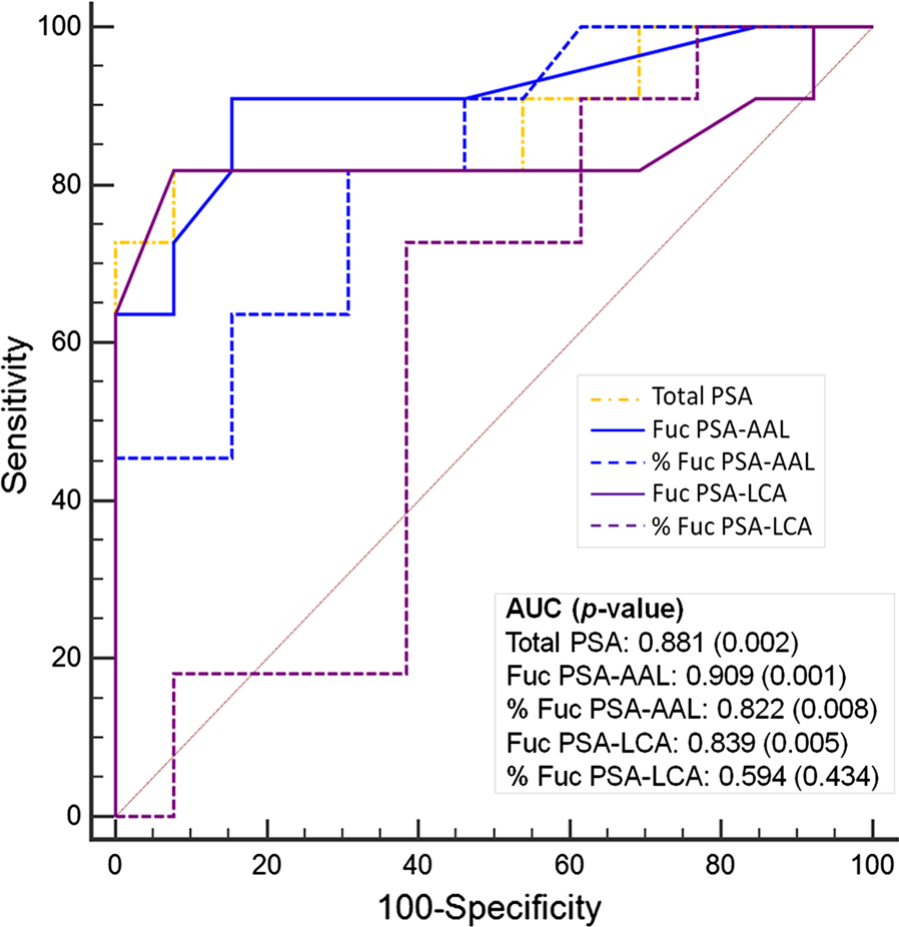
Receiver-operator characteristic (ROC) curves of serum total PSA, fucosylated PSA and % fucosylated PSA using AAL and LCA in separating Gleason pattern of (4 + 3) and above from Gleason pattern (6 or 3 + 4) on prostatectomy. Mann–Whitney U test was used for obtaining the *p* values

We analyzed fucosylated PSA–AAL, % fucosylated PSA–AAL, fucosylated PSA–LCA, % fucosylated PSA–LCA to differentiate the heterogeneity of pathological Gleason 7 (3 + 4) and (4 + 3) by receiver operating characteristic (ROC) curves analysis. Among them, fucosylated PSA–AAL had the best performance (AUC = 0.909), followed by total PSA (AUC = 0.881), fucosylated PSA–LCA (AUC = 0.839), % fucosylated PSA–AAL (AUC = 0.822), and % fucosylated PSA–LCA (AUC = 0.594) (Fig. 4). The differences among the AUCs of total PSA, fucosyalated PSA–AAL, % fucosylated PSA–AAL, % fucosylated PSA–AAL and fucosylated PSA–LCA were not statistically significant as it would be expected from the highly correlated nature between total PSA and fucosylated PSAs and the small number of samples in the current exploratory study.

To assess the potential clinical utility of the fucosylated PSA assays, we further investigated whether the addition of fucosylated PSA has the potential to provide additional information in the differentiation of Gleason score of 7 (4 + 3) and above with the Gleason scores of 6 and 7 (3 + 4). In multivariate logistic regression analysis that included total PSA, fucosylated PSA–LCA, and % fucosylated PSA–LCA, the *p* values were 0.118, 0.084, and 0.086, respectively, whereas in similar logistic regression analysis, total PSA, fucosylated PSA–AAL, and % fucosylated PSA–AAL, the *p* values were 0.375, 0.222, and 0.677, respectively. Furthermore, neither fucosylated PSA nor % fucosylated PSA alone was significant in logistic regression with total PSA.

## Discussion

The Gleason scoring system has been used to grade PCa to provide prognostic and risk assessment for PCa management. Gleason scores < 7 represent low risk, whereas a Gleason score between 7 and 10 suggests a higher risk and serves as a prognostic marker for progression. Among clinically diagnosed PCa patients, more than half of are diagnosed with Gleason 6 and lower, which is regarded as low-risk PCa and unlikely to cause significant symptoms or mortality. PCa with the Gleason score greater than 6, particularly those with the Gleason score greater than 7 is considered to be an AG PCa [33]. These patients have a greater risk of developing metastasis and other serious complications. Studies have shown that patients with Gleason 7 (4 + 3) tend to have a worse prognosis compared to patients with Gleason 7 (3 + 4) [34]. In order to determine the need for treatment, there is an urgent need to develop a strategy to differentiate aggressive from non-aggressive disease.

Our assays are based on lectin enrichment followed by immunoassay. We first captured fucosylated PSA in serum with lectins, then detected total and fucosylated PSA using the clinical Access Hybritech PSA assay. Two types of fucosylated PSA were studied, core Fuc and branch Fuc. LCA is the optimal lectin for selection of core Fuc whereas AAL selects both core Fuc and branch Fuc. Our results demonstrated that LCA showed a high percentage of binding than AAL. For the same PSA level, we detected more fucosylated PSA after LCA selection compared to AAL selection. However, compared patients with Gleason 6 (NAG PCa) with Gleason 7, and above (AG PCa), we did not find significant differences in fucosylated PSA captured with LCA (Mann–Whitney; *p *= 0.106). Li et al. has recently reported that serum fucosylated PSA–AAL and % fucosylated PSA–AAL could be predictive biomarkers to separate Gleason score > 6 tumors. Our results were consistent with the report. We also observed significant difference in serum % fucosylated PSA–AAL level when we combined the Gleason 7 (3 + 4) group with Gleason 6 and the Gleason 7 (4 + 3) group with Gleason 8, 9 (Mann–Whitney; *p *= 0.001). In multivariate logistic regression, the addition of fucosylated PSA and % fucosylated PSA to total PSA resulted improved separation of Gleason 7 (4 + 3) or above from Gleason 7 (3 + 4) or below. The *p* values indicated that their contributions were significant. However, when the fucosylated PSA or % fucosylated PSA was added to total PSA individually, neither of them was significant. For this exploratory study, the sample size was too small to draw definitive conclusions. However, this observation indicated the possibility that combined use of fucosylated PSA and % fucosylated PSA in combination could be used in place of total PSA with potentially improved performance identifying AG patients. Further studies with larger sample sizes will be needed to compare % fucosylated PSA values between AG and NAG patients to determine any statistical significance.

Several studies have reported elevated fucosylated PSA levels in AG PCa [25, 30]. Here, we developed fucosylated PSA immunoassays and applied to the analyses of PCa serum samples with high Gleason score using the AAL followed by clinical PSA test. This is consistent with our previous study using AAL and a PSA assay [25]. However, based on our results in this study, fucosylated PSA using LCA selection had high correlation with total PSA while the % fucosylated PSA–LCA selection could not differentiate between serum samples from high and low Gleason scores. This could be caused by different specificity of LCA and AAL to fucosylated glycans. AAL is known to bind both the branch and core Fuc while LCA is more specific toward to core-fucosylation. The elevated AAL binding from high Gleason score could be due to the different levels of branch Fuc.

## Conclusion

We have developed a new glycoproteomic strategy for serum fucosylated PSA quantitative detection. We have analyzed serum PSA levels and percentage of fucosylated PSA levels in men with PCa using lectin-affinity based clinical PSA immunoassays. Our data demonstrated that fucosylated PSA–AAL, % fucosylated PSA–AAL and fucosylated PSA–LCA level could be effective biomarkers to differentiate AG [especially for Gleason score = or > 7 (4 + 3)] from NAG PCa. We believe that a validation in a larger patient population is needed to confirm our promising results of the diagnostic values of fucosylated PSA–AAL and % fucosylated PSA–AAL for AG PCa.

## Abbreviations

PCa: prostate cancer
PSA: prostate-specific antigen
LCA: Lens culinaris agglutinin
AAL: Aleuria aurantia lectin
GS: Gleason score
FDA: Food and drug administration
Fuc: fucosylation
AG: aggressive
NAG: non-aggressive
BPH: benign prostatic hyperplasia
FUTs: fucosyltransferases
ISUP: International Society of Urological Pathology
